# Asymmetric controlled bidirectional remote preparation of two- and three-qubit equatorial state

**DOI:** 10.1038/s41598-018-37957-x

**Published:** 2019-02-14

**Authors:** Yi-Ru Sun, Xiu-Bo Chen, Gang Xu, Kai-Guo Yuan, Yi-Xian Yang

**Affiliations:** 1 Information Security Center, State Key Laboratory of Networking and Switching Technology, Beijing University of Posts and Telecommunications, Beijing, 100876 China; 2 GuiZhou University, State Key Laboratory of Public Big Data, Guizhou, Guiyang 550025 China

## Abstract

In this paper, a novel asymmetric controlled bidirectional remote preparation scheme is proposed. In our scheme, Alice and Bob are not only the senders but also the receivers with the control of Charlie. By using the eleven-qubit entangled state as the quantum channel, Alice prepares an arbitrary two-qubit equatorial state for Bob and Bob prepares an arbitrary three-qubit equatorial state for Alice simultaneously. Firstly, we give the construction process of the quantum channel. Secondly, the whole recovery operations are given. Alice and Bob can recover the prepared state determinately. Thirdly, we consider the effect of the noisy environment (amplitude-damping and phase-damping) in our scheme and calculate the fidelities of the output states. Finally, since our scheme does not need additional operations and auxiliary qubits, the efficiency of our scheme is higher than that of the previous schemes.

## Introduction

In quantum cryptography, quantum entanglement is magic, which plays a key role in many different types of communication schemes^1–3^. One of the applications is the remote state preparation (RSP)^4–6^. In a RSP scheme, the sender can prepare a known quantum state for the remote receiver through a pre-shared quantum channel and the appropriate measurements. After that, many various schemes were presented, such as controlled RSP (CRSP)^7^, joint RSP (JRSP)^8^.

However, the previous RSP schemes are unidirectional. The first controlled bidirectional RSP (CBRSP) scheme was presented by Cao *et al*.^9^ in 2014, where Alice and Bob send their single-qubit state to each other simultaneously. After that, in 2015, the deterministic, the probabilistic and the joint CBRSP schemes were devised by Sharma *et al*.^10^. And Peng *et al*.^11^ presented a five-party joint CBRSP scheme by using eight-qubit entangled state. In 2016, utilize the non-maximally and maximally six-qubit entangled state, Zhang *et al*.^12,13^ proposed two joint CBRSP schemes. In 2017, another five-party joint CBRSP scheme is proposed by Wang and Mo^14^ by using seven-qubit entangled state as the quantum channel. Sang^15^ presented a bidirectional controlled quantum information transmission scheme, where Alice teleports an arbitrary unknown single-qubit state to Bob and Bob remotely prepares an arbitrary known single-qubit state for Alice simultaneously. In 2018, a controlled bidirectional hybrid of remote state preparation and quantum teleportation scheme was proposed by Wu *et al*.^16^. By using thirteen-qubit entangled state as the quantum channel, Chen *et al*.^17^ presented a symmetric CBRSP scheme, where Alice and Bob prepared an arbitrary three-qubit state to each other simultaneously. Moreover, this scheme was discussed in four types of noisy environment and no auxiliary qubits are needed.

Moreover, many asymmetric CBRSP (ACBRSP) schemes have been proposed. In 2017, an ACBRSP scheme was presented by Sang *et al*.^18^, where Alice teleports an arbitrary unknown single-particle state to Bob and Bob remotely prepare an arbitrary known two-qubit state to Alice. Song *et al*.^19^ investigated an ACBRSP scheme of a single-qubit state and two-qubit state. Ma *et al*.^20^ proposed an ACBRSP scheme of an arbitrary four-qubit cluster-type state and a single-qubit state. In 2018, a scheme for bidirectional and hybrid quantum information transmission was proposed by Fang *et al*.^21^, where Alice can teleport an arbitrary single-qubit state (two-qubit state) to Bob and Bob can prepare a known two-qubit state (single-qubit state) to Alice simultaneously. In the previous schemes, many of them need the help of the auxiliary qubits and additional operations^19–21^. Furthermore, they do not give the construction process of the quantum channel. And only in the schemes^10,17^, the noise are considered. However, noise is a necessary factor that must be considered in actual communication. In ref.^22^, Zeng and Zhang have shown that the RSP scheme in real Hilbert space can only be implemented when the dimension of the space is 2, 4 or 8, while the equatorial case can be generalized without restriction on the dimension. Although the preparation of two states to each other can be completed by using two independent RSP schemes, simultaneity and fairness cannot be guaranteed in this process. However, in our scheme, the controlled bidirectional preparation of the equatorial state, Alice and Bob can transmit the prepared state to each other simultaneously with the control of Charlie. The simultaneity and fairness can be guaranteed in our scheme.

In this paper, a novel ACBRSP scheme is put forward. With the control of Charlie, Alice prepares an arbitrary two-qubit equatorial state for Bob and Bob prepares an arbitrary three-qubit equatorial state for Alice at the same time. We firstly generate the eleven-qubit entangled state as the quantum channel. Then, through the appropriate measurements and the recovery operations, Alice and Bob can reconstruct the prepared state determinately. Moreover, we consider the effect of the noisy environment in our scheme and calculate the fidelities of the output states. Last but not the least, some discussions and conclusions are given. The result shows that our scheme does not need additional operations and auxiliary qubits. And the prepared states are the arbitrary two- and three-qubit states, so the efficiency of our scheme is higher than that of the previous schemes.

## Results

### The construction process of the quantum channel

We use the eleven-qubit product state as an input state^23^ and implement the *Hadamard* (*H*) and *CNOT* operations to construct the eleven-qubit entangled state as the quantum channel^24^. The process of the construction of the quantum channel is given as follows.

The input state is the eleven-qubit product state $|{\phi }_{1}\rangle$ as

1 $$|{\phi }_{1}\rangle ={|0\rangle }_{1}{|0\rangle }_{2}{|0\rangle }_{3}{|0\rangle }_{4}{|0\rangle }_{5}{|0\rangle }_{6}{|0\rangle }_{7}{|0\rangle }_{8}{|0\rangle }_{9}{|0\rangle }_{a}{|0\rangle }_{b}.$$

**C1** We implement the *H* operation on qubit 1 and rewrite the $|{\phi }_{1}\rangle$ as

2 $$|{\phi }_{2}\rangle =\frac{1}{\sqrt{2}}{(|0\rangle +|1\rangle )}_{1}{|0\rangle }_{2}{|0\rangle }_{3}{|0\rangle }_{4}{|0\rangle }_{5}{|0\rangle }_{6}{|0\rangle }_{7}{|0\rangle }_{8}{|0\rangle }_{9}{|0\rangle }_{a}{|0\rangle }_{b}.$$

**C2** Operate *CNOT* operations on the qubit pairs (1, 3), (1, 5), (1, 7), (1, 9), (1, *b*), respectively, where qubit 1 is used as controlled qubit and each of five qubits 3, 5, 7, 9, *b* are used as target qubit. We rewrite the $|{\phi }_{2}\rangle$ as

3 $$|{\phi }_{3}\rangle =\frac{1}{\sqrt{2}}{(|0\rangle |0000000000\rangle +|1\rangle |0101010101\rangle )}_{123456789ab}\,.$$

**C3** After implementing the *H* operations on the qubits 2, 4, 6, 8, *a*, we execute *CNOT* operations on the qubit pairs (2, 3), (4, 5), (6, 7), (8, 9), (*a*, *b*), respectively, where qubits 2, 4, 6, 8, *a* are used as controlled qubit and each of five qubits 3, 5, 7, 9, *b* is used as target qubit. The eleven-qubit entangled state can be generated as

4 $$|{\phi }_{4}\rangle =\frac{1}{\sqrt{2}}({|0\rangle }_{1}{|{{\rm{\Phi }}}^{+}\rangle }_{23}{|{{\rm{\Phi }}}^{+}\rangle }_{45}{|{{\rm{\Phi }}}^{+}\rangle }_{67}{|{{\rm{\Phi }}}^{+}\rangle }_{89}{|{{\rm{\Phi }}}^{+}\rangle }_{ab}+{|1\rangle }_{1}{|{{\rm{\Psi }}}^{+}\rangle }_{23}{|{{\rm{\Psi }}}^{+}\rangle }_{45}{|{{\rm{\Psi }}}^{+}\rangle }_{67}{|{{\rm{\Psi }}}^{+}\rangle }_{89}{|{{\rm{\Psi }}}^{+}\rangle }_{ab}),$$

where $$|{{\rm{\Phi }}}^{+}\rangle =\frac{1}{\sqrt{2}}(|00\rangle +|11\rangle )$$ and $$|{{\rm{\Psi }}}^{+}\rangle =\frac{1}{\sqrt{2}}(|01\rangle +|10\rangle )$$.

**Figure 1:**
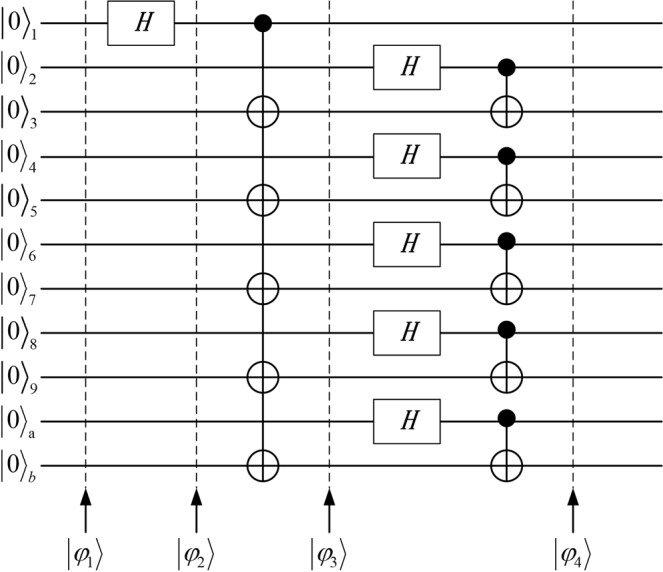
The construction of the quantum channel.

In Fig. 1, we give the circuit diagram of the construction of the quantum channel.

### The ACBRSP scheme of an arbitrary two- and three-qubit equatorial state

By using eleven-qubit entangled state $|{\phi }_{4}\rangle$ as the quantum channel, we propose a novel ACBRSP scheme. In the scheme, with the control of Charlie, Alice prepares an arbitrary two-qubit equatorial state for Bob and Bob prepares an arbitrary three-qubit equatorial state for Alice. They pre-share this quantum channel at first which is constructed by Charlie. Then, Alice and Bob can complete the preparation task through the appropriate measurements and the corresponding recovery operations.

In the scheme, as an honest controller, Charlie constructs the eleven-qubit entangled state as the quantum channel and holds qubit 1 in his hand. Then, he sends the qubits 2, 4, 6, 8, *a* to Alice and sends the qubits 3, 5, 7, 9, *b* to Bob. Before distributing these qubits, the participants have to check eavesdropping by using the decoy qubits to ensure the security of the distribution. The process of checking eavesdropping is the same as that in ref.^17^. After successfully passing the check eavesdropping, the quantum channel can be securely shared by Alice, Bob and Charlie. Next, we will describe our ACBRSP scheme in detail.

**A1** They pre-share the quantum channel $|{\phi }_{4}\rangle$. Alice wants to prepare a two-qubit equatorial state $|{\varphi }_{A}\rangle$ for Bob and Bob wants to prepare a three-qubit equatorial state $|{\varphi }_{B}\rangle$ for Alice, where

5 $$|{\varphi }_{A}\rangle =\frac{1}{2}(|00\rangle +{e}^{i{\theta }_{1}}|01\rangle +{e}^{i{\theta }_{2}}|10\rangle +{e}^{i{\theta }_{3}}|11\rangle ),$$

6 $$|{\varphi }_{B}\rangle =\frac{1}{2\sqrt{2}}(|000\rangle \,+\,{e}^{i{\delta }_{1}}|001\rangle \,+\,{e}^{i{\delta }_{2}}|010\rangle \,+\,{e}^{i{\delta }_{3}}|011\rangle \,+\,{e}^{i{\delta }_{4}}|100\rangle \,+\,{e}^{i{\delta }_{5}}|101\rangle \,+\,{e}^{i{\delta }_{6}}|110\rangle \,+\,{e}^{i{\delta }_{7}}|111\rangle ),$$

Here, ${\theta }_{1},{\theta }_{2},{\theta }_{3},{\delta }_{k}$ are real and $0\le {\theta }_{1},{\theta }_{2},{\theta }_{3},{\delta }_{k}\le 2\pi$, $k=1,2,\mathrm{...},7$.

**A2** Alice carries out two-qubit measurement on her qubits 2, 4. The measurement basis is ${(|{A}_{0}\rangle ,|{A}_{1}\rangle ,|{A}_{2}\rangle ,|{A}_{3}\rangle )}_{24}$, where

7 $$(\begin{array}{c}|{A}_{0}\rangle \\ |{A}_{1}\rangle \\ |{A}_{2}\rangle \\ |{A}_{3}\rangle \end{array})=\frac{1}{2}(\begin{array}{cccc}1 & {e}^{-i{\theta }_{1}} & {e}^{-i{\theta }_{2}} & {e}^{-i{\theta }_{3}}\\ 1 & -{e}^{-i{\theta }_{1}} & {e}^{-i{\theta }_{2}} & -{e}^{-i{\theta }_{3}}\\ 1 & i{e}^{-i{\theta }_{1}} & -{e}^{-i{\theta }_{2}} & -i{e}^{-i{\theta }_{3}}\\ 1 & -i{e}^{-i{\theta }_{1}} & -{e}^{-i{\theta }_{2}} & i{e}^{-i{\theta }_{3}}\end{array})(\begin{array}{c}|00\rangle \\ |01\rangle \\ |10\rangle \\ |11\rangle \end{array}),$$

Bob performs three-qubit measurement on his qubits 7, 9, *b*. The measurement basis is $(|{B}_{0}\rangle ,|{B}_{1}\rangle ,|{B}_{2}\rangle ,|{B}_{3}\rangle ,$ ${|{B}_{4}\rangle ,|{B}_{5}\rangle ,|{B}_{6}\rangle ,|{B}_{7}\rangle )}_{79b}$ as

8 $$\begin{array}{c}{(|{B}_{0}\rangle ,|{B}_{1}\rangle ,|{B}_{2}\rangle ,|{B}_{3}\rangle ,|{B}_{4}\rangle ,|{B}_{5}\rangle ,|{B}_{6}\rangle ,|{B}_{7}\rangle )}_{79b}^{T}\\ \,=\,B{(|000\rangle ,|001\rangle ,|010\rangle ,|011\rangle ,|100\rangle ,|101\rangle ,|110\rangle ,|111\rangle )}_{79b}^{T}\end{array}$$

where

$$B=\frac{1}{2\sqrt{2}}(\begin{array}{cccccccc}1 & {e}^{-i{\delta }_{1}} & {e}^{-i{\delta }_{2}} & {e}^{-i{\delta }_{3}} & {e}^{-i{\delta }_{4}} & {e}^{-i{\delta }_{5}} & {e}^{-i{\delta }_{6}} & {e}^{-i{\delta }_{7}}\\ 1 & -{e}^{-i{\delta }_{1}} & {e}^{-i{\delta }_{2}} & -{e}^{-i{\delta }_{3}} & {e}^{-i{\delta }_{4}} & -{e}^{-i{\delta }_{5}} & {e}^{-i{\delta }_{6}} & -{e}^{-i{\delta }_{7}}\\ 1 & i{e}^{-i{\delta }_{1}} & -{e}^{-i{\delta }_{2}} & -i{e}^{-i{\delta }_{3}} & {e}^{-i{\delta }_{4}} & i{e}^{-i{\delta }_{5}} & -{e}^{-i{\delta }_{6}} & -i{e}^{-i{\delta }_{7}}\\ 1 & -i{e}^{-i{\delta }_{1}} & -{e}^{-i{\delta }_{2}} & i{e}^{-i{\delta }_{3}} & {e}^{-i{\delta }_{4}} & -i{e}^{-i{\delta }_{5}} & -{e}^{-i{\delta }_{6}} & i{e}^{-i{\delta }_{7}}\\ 1 & {e}^{\pi i/4}{e}^{-i{\delta }_{1}} & i{e}^{-i{\delta }_{2}} & i{e}^{\pi i/4}{e}^{-i{\delta }_{3}} & -{e}^{-i{\delta }_{4}} & -{e}^{\pi i/4}{e}^{-i{\delta }_{5}} & -i{e}^{-i{\delta }_{6}} & -i{e}^{\pi i/4}{e}^{-i{\delta }_{7}}\\ 1 & -{e}^{\pi i/4}{e}^{-i{\delta }_{1}} & i{e}^{-i{\delta }_{2}} & -i{e}^{\pi i/4}{e}^{-i{\delta }_{3}} & -{e}^{-i{\delta }_{4}} & {e}^{\pi i/4}{e}^{-i{\delta }_{5}} & -i{e}^{-i{\delta }_{6}} & i{e}^{\pi i/4}{e}^{-i{\delta }_{7}}\\ 1 & i{e}^{\pi i/4}{e}^{-i{\delta }_{1}} & -i{e}^{-i{\delta }_{2}} & {e}^{\pi i/4}{e}^{-i{\delta }_{3}} & -{e}^{-i{\delta }_{4}} & -i{e}^{\pi i/4}{e}^{-i{\delta }_{5}} & i{e}^{-i{\delta }_{6}} & -{e}^{\pi i/4}{e}^{-i{\delta }_{7}}\\ 1 & -i{e}^{\pi i/4}{e}^{-i{\delta }_{1}} & -i{e}^{-i{\delta }_{2}} & -{e}^{\pi i/4}{e}^{-i{\delta }_{3}} & -{e}^{-i{\delta }_{4}} & i{e}^{\pi i/4}{e}^{-i{\delta }_{5}} & i{e}^{-i{\delta }_{6}} & {e}^{\pi i/4}{e}^{-i{\delta }_{7}}\end{array})$$

The quantum channel $|{\phi }_{4}\rangle$ becomes:

9 $$\begin{array}{rcl}|{\phi }_{4}\rangle  & = & 1/8\{{|0\rangle }_{1}1/2[{|{A}_{0}\rangle }_{24}{(|00\rangle +{e}^{i{\theta }_{1}}|01\rangle +{e}^{i{\theta }_{2}}|10\rangle +{e}^{i{\theta }_{3}}|11\rangle )}_{35}\\  &  & +{|{A}_{1}\rangle }_{24}{(|00\rangle -{e}^{i{\theta }_{1}}|01\rangle +{e}^{i{\theta }_{2}}|10\rangle -{e}^{i{\theta }_{3}}|11\rangle )}_{35}+{|{A}_{2}\rangle }_{24}(|00\rangle \\  &  & -i{e}^{i{\theta }_{1}}|01\rangle -{e}^{i{\theta }_{2}}|10\rangle +i{e}^{i{\theta }_{3}}|11\rangle {)}_{35}+{|{A}_{3}\rangle }_{24}(|00\rangle +i{e}^{i{\theta }_{1}}|01\rangle \\  &  & -{e}^{i{\theta }_{2}}|10\rangle -i{e}^{i{\theta }_{3}}|11\rangle {)}_{35}](|{B}_{0}\rangle |{C}_{0}\rangle +|{B}_{1}\rangle |{C}_{1}\rangle +|{B}_{2}\rangle |{C}_{2}\rangle \\  &  & +|{B}_{3}\rangle |{C}_{3}\rangle +|{B}_{4}\rangle |{C}_{4}\rangle +|{B}_{5}\rangle |{C}_{5}\rangle +|{B}_{6}\rangle |{C}_{6}\rangle +|{B}_{7}\rangle |{C}_{7}\rangle {)}_{79b68a}\\  &  & +{|1\rangle }_{1}1/2[{|{A}_{0}\rangle }_{24}{(|11\rangle +{e}^{i{\theta }_{1}}|10\rangle +{e}^{i{\theta }_{2}}|01\rangle +{e}^{i{\theta }_{3}}|00\rangle )}_{35}\\  &  & +{|{A}_{1}\rangle }_{24}{(|11\rangle -{e}^{i{\theta }_{1}}|10\rangle +{e}^{i{\theta }_{2}}|01\rangle -{e}^{i{\theta }_{3}}|00\rangle )}_{35}\\  &  & +{|{A}_{2}\rangle }_{24}{(|11\rangle -i{e}^{i{\theta }_{1}}|10\rangle -{e}^{i{\theta }_{2}}|01\rangle +i{e}^{i{\theta }_{3}}|00\rangle )}_{35}\\  &  & +{|{A}_{3}\rangle }_{24}(|11\rangle +i{e}^{i{\theta }_{1}}|10\rangle -{e}^{i{\theta }_{2}}|01\rangle -i{e}^{i{\theta }_{3}}|00\rangle {)}_{35}](|{B}_{0}\rangle |{D}_{0}\rangle \\  &  & +|{B}_{1}\rangle |{D}_{1}\rangle +|{B}_{2}\rangle |{D}_{2}\rangle +|{B}_{3}\rangle |{D}_{3}\rangle +|{B}_{4}\rangle |{D}_{4}\rangle \\  &  & +|{B}_{5}\rangle |{D}_{5}\rangle +|{B}_{6}\rangle |{D}_{6}\rangle +|{B}_{7}\rangle |{D}_{7}\rangle {)}_{79b68a}\},\end{array}$$

where

$$\begin{array}{rcl}|{C}_{0}\rangle  & = & \frac{1}{2\sqrt{2}}(|000\rangle +{e}^{i{\delta }_{1}}|001\rangle +{e}^{i{\delta }_{2}}|010\rangle +{e}^{i{\delta }_{3}}|011\rangle \\  &  & +{e}^{i{\delta }_{4}}|100\rangle +{e}^{i{\delta }_{5}}|101\rangle +{e}^{i{\delta }_{6}}|110\rangle +{e}^{i{\delta }_{7}}|111\rangle )\\ |{C}_{1}\rangle  & = & \frac{1}{2\sqrt{2}}(|000\rangle -{e}^{i{\delta }_{1}}|001\rangle +{e}^{i{\delta }_{2}}|010\rangle -{e}^{i{\delta }_{3}}|011\rangle \\  &  & +{e}^{i{\delta }_{4}}|100\rangle -{e}^{i{\delta }_{5}}|101\rangle +{e}^{i{\delta }_{6}}|110\rangle -{e}^{i{\delta }_{7}}|111\rangle )\\ |{C}_{2}\rangle  & = & \frac{1}{2\sqrt{2}}(|000\rangle +i{e}^{i{\delta }_{1}}|001\rangle -{e}^{i{\delta }_{2}}|010\rangle -i{e}^{i{\delta }_{3}}|011\rangle \\  &  & +{e}^{i{\delta }_{4}}|100\rangle +i{e}^{i{\delta }_{5}}|101\rangle -{e}^{i{\delta }_{6}}|110\rangle -i{e}^{i{\delta }_{7}}|111\rangle )\\ |{C}_{3}\rangle  & = & \frac{1}{2\sqrt{2}}(|000\rangle -i{e}^{i{\delta }_{1}}|001\rangle -{e}^{i{\delta }_{2}}|010\rangle +i{e}^{i{\delta }_{3}}|011\rangle \\  &  & +{e}^{i{\delta }_{4}}|100\rangle -i{e}^{i{\delta }_{5}}|101\rangle -{e}^{i{\delta }_{6}}|110\rangle +i{e}^{i{\delta }_{7}}|111\rangle )\\ |{C}_{4}\rangle  & = & \frac{1}{2\sqrt{2}}(|000\rangle +{e}^{i{\delta }_{1}-\frac{\pi i}{4}}|001\rangle -i{e}^{i{\delta }_{2}}|010\rangle -i{e}^{i{\delta }_{3}-\frac{\pi i}{4}}|011\rangle \\  &  & -{e}^{i{\delta }_{4}}|100\rangle -{e}^{i{\delta }_{5}-\frac{\pi i}{4}}|101\rangle +i{e}^{i{\delta }_{6}}|110\rangle +i{e}^{i{\delta }_{7}-\frac{\pi i}{4}}|111\rangle )\\ |{C}_{5}\rangle  & = & \frac{1}{2\sqrt{2}}(|000\rangle -{e}^{i{\delta }_{1}-\frac{\pi i}{4}}|001\rangle -i{e}^{i{\delta }_{2}}|010\rangle +i{e}^{i{\delta }_{3}-\frac{\pi i}{4}}|011\rangle \\  &  & -{e}^{i{\delta }_{4}}|100\rangle +{e}^{i{\delta }_{5}-\frac{\pi i}{4}}|101\rangle +i{e}^{i{\delta }_{6}}|110\rangle -i{e}^{i{\delta }_{7}-\frac{\pi i}{4}}|111\rangle )\\ |{C}_{6}\rangle  & = & \frac{1}{2\sqrt{2}}(|000\rangle +i{e}^{i{\delta }_{1}-\frac{\pi i}{4}}|001\rangle +i{e}^{i{\delta }_{2}}|010\rangle -{e}^{i{\delta }_{3}-\frac{\pi i}{4}}|011\rangle \\  &  & -{e}^{i{\delta }_{4}}|100\rangle -i{e}^{i{\delta }_{5}-\frac{\pi i}{4}}|101\rangle -i{e}^{i{\delta }_{6}}|110\rangle +{e}^{i{\delta }_{7}-\frac{\pi i}{4}}|111\rangle )\\ |{C}_{7}\rangle  & = & \frac{1}{2\sqrt{2}}(|000\rangle -i{e}^{i{\delta }_{1}-\frac{\pi i}{4}}|001\rangle +i{e}^{i{\delta }_{2}}|010\rangle \\  &  & +{e}^{i{\delta }_{3}-\frac{\pi i}{4}}|011\rangle -{e}^{i{\delta }_{4}}|100\rangle +i{e}^{i{\delta }_{5}-\frac{\pi i}{4}}|101\rangle -i{e}^{i{\delta }_{6}}|110\rangle -{e}^{i{\delta }_{7}-\frac{\pi i}{4}}|111\rangle )\\ |{D}_{0}\rangle  & = & \frac{1}{2\sqrt{2}}(|111\rangle +{e}^{i{\delta }_{1}}|101\rangle +{e}^{i{\delta }_{2}}|011\rangle \\  &  & +{e}^{i{\delta }_{3}}|001\rangle +{e}^{i{\delta }_{4}}|110\rangle +{e}^{i{\delta }_{5}}|100\rangle +{e}^{i{\delta }_{6}}|010\rangle +{e}^{i{\delta }_{7}}|000\rangle )\\ |{D}_{1}\rangle  & = & \frac{1}{2\sqrt{2}}(|111\rangle -{e}^{i{\delta }_{1}}|101\rangle +{e}^{i{\delta }_{2}}|011\rangle \\  &  & -{e}^{i{\delta }_{3}}|001\rangle +{e}^{i{\delta }_{4}}|110\rangle -{e}^{i{\delta }_{5}}|100\rangle +{e}^{i{\delta }_{6}}|010\rangle -{e}^{i{\delta }_{7}}|000\rangle )\\ |{D}_{2}\rangle  & = & \frac{1}{2\sqrt{2}}(|111\rangle +i{e}^{i{\delta }_{1}}|101\rangle -{e}^{i{\delta }_{2}}|011\rangle \\  &  & -i{e}^{i{\delta }_{3}}|001\rangle +{e}^{i{\delta }_{4}}|110\rangle +i{e}^{i{\delta }_{5}}|100\rangle -{e}^{i{\delta }_{6}}|010\rangle -i{e}^{i{\delta }_{7}}|000\rangle )\\ |{D}_{3}\rangle  & = & \frac{1}{2\sqrt{2}}(|111\rangle -i{e}^{i{\delta }_{1}}|101\rangle -{e}^{i{\delta }_{2}}|011\rangle +i{e}^{i{\delta }_{3}}|001\rangle \\  &  & +{e}^{i{\delta }_{4}}|110\rangle -i{e}^{i{\delta }_{5}}|100\rangle -{e}^{i{\delta }_{6}}|010\rangle +i{e}^{i{\delta }_{7}}|000\rangle )\\ |{D}_{4}\rangle  & = & \frac{1}{2\sqrt{2}}(|111\rangle +{e}^{i{\delta }_{1}-\frac{\pi i}{4}}|101\rangle -i{e}^{i{\delta }_{2}}|011\rangle -i{e}^{i{\delta }_{3}-\frac{\pi i}{4}}|001\rangle \\  &  & -{e}^{i{\delta }_{4}}|110\rangle -{e}^{i{\delta }_{5}-\frac{\pi i}{4}}|100\rangle +i{e}^{i{\delta }_{6}}|010\rangle +i{e}^{i{\delta }_{7}-\frac{\pi i}{4}}|000\rangle )\\ {|{D}_{5}\rangle }_{68a} & = & \frac{1}{2\sqrt{2}}(|111\rangle -{e}^{i{\delta }_{1}-\frac{\pi i}{4}}|101\rangle -i{e}^{i{\delta }_{2}}|011\rangle +i{e}^{i{\delta }_{3}-\frac{\pi i}{4}}|001\rangle \\  &  & -{e}^{i{\delta }_{4}}|110\rangle +{e}^{i{\delta }_{5}-\frac{\pi i}{4}}|100\rangle +i{e}^{i{\delta }_{6}}|010\rangle -i{e}^{i{\delta }_{7}-\frac{\pi i}{4}}|000\rangle )\\ {|{D}_{6}\rangle }_{68a} & = & \frac{1}{2\sqrt{2}}(|111\rangle +i{e}^{i{\delta }_{1}-\frac{\pi i}{4}}|101\rangle +i{e}^{i{\delta }_{2}}|011\rangle -{e}^{i{\delta }_{3}-\frac{\pi i}{4}}|001\rangle \\  &  & -{e}^{i{\delta }_{4}}|110\rangle -i{e}^{i{\delta }_{5}-\frac{\pi i}{4}}|100\rangle -i{e}^{i{\delta }_{6}}|010\rangle +{e}^{i{\delta }_{7}-\frac{\pi i}{4}}|000\rangle )\\ {|{D}_{7}\rangle }_{68a} & = & \frac{1}{2\sqrt{2}}(|111\rangle -i{e}^{i{\delta }_{1}-\frac{\pi i}{4}}|101\rangle +i{e}^{i{\delta }_{2}}|011\rangle +{e}^{i{\delta }_{3}-\frac{\pi i}{4}}|001\rangle \\  &  & -{e}^{i{\delta }_{4}}|110\rangle +i{e}^{i{\delta }_{5}-\frac{\pi i}{4}}|100\rangle -i{e}^{i{\delta }_{6}}|010\rangle -{e}^{i{\delta }_{7}-\frac{\pi i}{4}}|000\rangle )\end{array}$$

**A3** Alice’s and Bob’s measurement results are ${|{A}_{s}\rangle }_{24},{|{B}_{t}\rangle }_{79b}$, $0\le s\le 3$ and $0\le t\le 7$. They send their measurement results to others. After that, Charlie implements a single-qubit measurement in the basis $\{|0\rangle ,|1\rangle \}$ on qubit 1 and sends the result to Alice and Bob through the classical channel.

**Figure 2:**
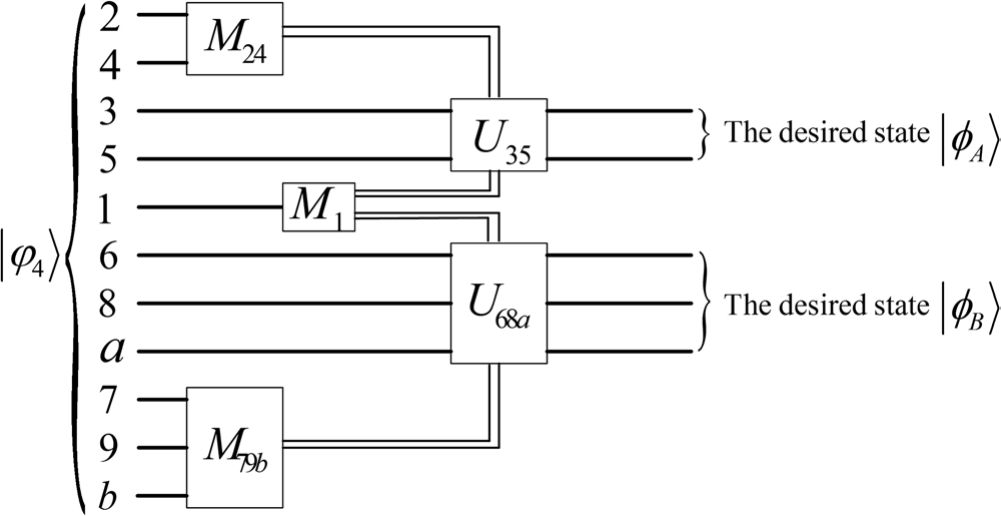
The process of the ACBRSP scheme. The ${M}_{24},{M}_{79b},{M}_{1}$ are short for the measurements of Alice, Bob and Charlie on their own qubits, respectively. ${U}_{35},{U}_{68a}$ are the recovery operations of Bob and Alice, respectively.

**A4** In the end, after receiving others’ measurement results, Alice can recover the three-qubit equatorial state $|{\varphi }_{B}\rangle$ by applying the corresponding recovery operations on her qubits 6, 8, *a* and Bob can recover the two-qubit equatorial state $|{\varphi }_{A}\rangle$ by applying the corresponding recovery operations on his qubits 3, 5. The process of the scheme is given in Fig. 2.

For instance, we assume Alice’s and Bob’s measurement results are ${|{A}_{0}\rangle }_{24},{|{B}_{0}\rangle }_{79b}$. If Charlie’s measurement result is ${|0\rangle }_{1}$, Alice (Bob) recovers the prepared state ${|{\varphi }_{B}\rangle }_{68a}$(${|{\varphi }_{A}\rangle }_{35}$) by performing the recovery operations ${I}_{6}\otimes {I}_{8}\otimes {I}_{a}$ (${I}_{3}\otimes {I}_{5}$) on the qubits 6, 8, *a* (3, 5), respectively. If Charlie’s measurement result is ${|1\rangle }_{1}$, Alice’s (Bob’s) recovery operations are ${X}_{6}\otimes {X}_{8}\otimes {X}_{a}$ (${X}_{3}\otimes {X}_{5}$), respectively. For other measurement results, Alice and Bob can also use corresponding recovery operation to get the prepared state. All the measurement results and corresponding recovery operations are given in Supplementary information. Furthermore, the scheme is completed deterministically.

### The ACBRSP scheme in amplitude-damping and phase-damping noisy environment

It is impossible to have a noiseless communication environment in practice. For example, Sharma *et al*.^10^ discussed the effects of two well-known noise processes, the amplitude-damping and phase-damping noise, in their scheme. In this section, we will consider the ACBRSP scheme in two types of noisy environment (amplitude-damping and phase-damping).

In ACBRSP scheme, Charlie constructs the quantum channel $|{\phi }_{4}\rangle$ and holds the qubit 1. Since the qubit 1 is not transmitted in the noisy environment, so it is not affected by the noise. Charlie sends the qubits 2, 4, 6, 8, *a* (3, 5, 7, 9, *b*) to Alice (Bob) via the channel-I (channel-II). We assume that the channel-I and channel-II are in the same noisy environment. Therefore, the qubits 2, 4, 6, 8, *a* (3, 5, 7, 9, *b*) are affected by the same Kraus operator. The density matrix of the quantum channel $|{\phi }_{4}\rangle$ can be calculated as $\rho =|{\phi }_{4}\rangle \langle {\phi }_{4}|$.

#### The Kraus operators

In the amplitude-damping noisy environment, the Kraus operators^25^ are

10 $${E}_{0}^{A}=(\begin{array}{cc}1 & 0\\ 0 & \sqrt{1-{p}_{a}}\end{array}),\,{E}_{1}^{A}=(\begin{array}{cc}0 & \sqrt{{p}_{a}}\\ 0 & 0\end{array}),$$

where ${p}_{a}\,(0\le {p}_{a}\le 1)$ is the decoherence rate. It is the probability of missing a photon. Because of the interaction with this noisy environment, a system undergoes energy dissipation.

In phase-damping noisy environment, it describes the loss of information about the relative phase in the quantum state. The Kraus operators^25^ are

11 $${E}_{0}^{P}=(\begin{array}{cc}\sqrt{1-{p}_{p}} & 0\\ 0 & \sqrt{1-{p}_{p}}\end{array}),\,{E}_{1}^{P}=(\begin{array}{cc}\sqrt{{p}_{p}} & 0\\ 0 & 0\end{array}),\,{E}_{2}^{P}=(\begin{array}{cc}0 & 0\\ 0 & \sqrt{{p}_{p}}\end{array}),$$

where ${p}_{p}\,(0\le {p}_{p}\le 1)$ is the decoherence rate.

#### The output state

The qubits 2, 4, 6, 8, *a* (3, 5, 7, 9, *b*) are transmitted in the amplitude-damping and phase-damping noisy environment, we rewrite the density matrix $\rho$ as ${\varepsilon }^{A}(\rho )$ and ${\varepsilon }^{P}(\rho )$.

12 $$\begin{array}{rcl}{\varepsilon }^{A}{(\rho )}_{1352468a79b} & = & \sum _{m,n=0,1}{I}_{1}{({E}_{m}^{A})}_{3}{({E}_{m}^{A})}_{5}{({E}_{n}^{A})}_{2}{({E}_{n}^{A})}_{4}{({E}_{n}^{A})}_{6}{({E}_{n}^{A})}_{8}{({E}_{n}^{A})}_{a}{({E}_{m}^{A})}_{7}{({E}_{m}^{A})}_{9}{({E}_{m}^{A})}_{b}\rho \\  &  & [{I}_{1}{({E}_{m}^{A})}_{3}{({E}_{m}^{A})}_{5}{({E}_{n}^{A})}_{2}{({E}_{n}^{A})}_{4}{({E}_{n}^{A})}_{6}{({E}_{n}^{A})}_{8}{({E}_{n}^{A})}_{a}{({E}_{m}^{A})}_{7}\\  &  & {({E}_{m}^{A})}_{9}{({E}_{m}^{A})}_{b}{]}^{\dagger }=\frac{1}{64}\{{|0\rangle }_{1}[|0000\rangle +(1-{p}_{a})|0101\rangle \\  &  & +\,(1-{p}_{a})|1010\rangle +{(1-{p}_{a})}^{2}|1111\rangle ]\times [|000000\rangle \\  &  & +\,(1-{p}_{a})|001001\rangle +(1-{p}_{a})|010010\rangle +{(1-{p}_{a})}^{2}\\  &  & \times \,|011011\rangle +(1-{p}_{a})|100100\rangle +{(1-{p}_{a})}^{2}|101101\rangle +{(1-{p}_{a})}^{2}\\  &  & \times \,|110110\rangle +{(1-{p}_{a})}^{3}|111111\rangle ]+{|1\rangle }_{1}(1-{p}_{a})[|1100\rangle +|1001\rangle +|0110\rangle \\  &  & +\,|0011\rangle {]}_{3524}{(\sqrt{1-{p}_{a}})}^{3}[vz|111000\rangle +|110001\rangle +|101010\rangle +|100011\rangle ]\}\\  &  & +\,|011100\rangle +|010101\rangle +|001110\rangle +|000111\rangle ]\}\{{\langle 0|}_{1}[\langle 0000|+(1-{p}_{a})\langle 0101|\\  &  & +\,(1-{p}_{a})\langle 1010|+{(1-{p}_{a})}^{2}\langle 1111|][\langle 000000|+(1-{p}_{a})\langle 001001|\\  &  & +\,(1-{p}_{a})\langle 010010|+{(1-{p}_{a})}^{2}\langle 011011|+(1-{p}_{a})\langle 100100|\\  &  & +{(1-{p}_{a})}^{2}\langle 101101|+{(1-{p}_{a})}^{2}\langle 110110|+{(1-{p}_{a})}^{3}\langle 111111|]\\  &  & +\,{\langle 1|}_{1}(1-{p}_{a})[\langle 1100|+\langle 1001|+\langle 0110|+\langle 0011|]{(\sqrt{1-{p}_{a}})}^{3}[\langle 111000|\\  &  & +\langle 110001|+\langle 101010|+\langle 100011|+\langle 011100|+\langle 010101|+\langle 001110|\\  &  & +\,\langle 000111|]+[{|0\rangle }_{1}\sqrt{{p}_{a}^{5}}{(\sqrt{1-{p}_{a}})}^{5}|0011\rangle |111000\rangle +{|1\rangle }_{1}\sqrt{{p}_{a}^{5}}|0000\rangle |000000\rangle ]\\  &  & \times \,[{\langle 0|}_{1}\sqrt{{p}_{a}^{5}}{(\sqrt{1-{p}_{a}})}^{5}\langle 0011|\langle 111000|+{\langle 1|}_{1}\sqrt{{p}_{a}^{5}}\langle 0000|\langle 000000|]\\  &  & +\,[{|0\rangle }_{1}\sqrt{{p}_{a}^{5}}{(\sqrt{1-{p}_{a}})}^{5}|1100\rangle |000111\rangle +{|1\rangle }_{1}\sqrt{{p}_{a}^{5}}|0000\rangle |000000\rangle ]\\  &  & [{\langle 0|}_{1}\sqrt{{p}_{a}^{5}}{(\sqrt{1-{p}_{a}})}^{5}\langle 1100|\langle 000111|+{\langle 1|}_{1}\sqrt{{p}_{a}^{5}}{(\sqrt{1-{p}_{a}})}^{5}\langle 0000|\langle 000000|]\\  &  & +({|0\rangle }_{1}{p}_{a}^{5}|0000\rangle |000000\rangle )({\langle 0|}_{1}{p}_{a}^{5}\langle 0000|\langle 000000|)\}.\end{array}$$

13 $$\begin{array}{rcl}{\varepsilon }^{P}{(\rho )}_{1352468a79b} & = & \sum _{m,n=0,1,2}{I}_{1}{({E}_{m}^{P})}_{3}{({E}_{m}^{P})}_{5}{({E}_{n}^{P})}_{2}{({E}_{n}^{P})}_{4}{({E}_{n}^{P})}_{6}{({E}_{n}^{P})}_{8}{({E}_{n}^{P})}_{a}{({E}_{m}^{P})}_{7}{({E}_{m}^{P})}_{9}{({E}_{m}^{P})}_{b}\rho \\  &  & {[{I}_{1}{({E}_{m}^{P})}_{3}{({E}_{m}^{P})}_{5}{({E}_{n}^{P})}_{2}{({E}_{n}^{P})}_{4}{({E}_{n}^{P})}_{6}{({E}_{n}^{P})}_{8}{({E}_{n}^{P})}_{a}{({E}_{m}^{P})}_{7}{({E}_{m}^{P})}_{9}{({E}_{m}^{P})}_{b}]}^{\dagger }\\  & = & \frac{1}{64}\{{|0\rangle }_{1}{(1-{p}_{p})}^{5}[|0000\rangle +|0101\rangle +|1010\rangle +|1111\rangle ]\\  &  & \times \,[|000000\rangle +|001001\rangle +\,|010010\rangle +|011011\rangle \\  &  & +\,|100100\rangle +|101101\rangle +|110110\rangle +|111111\rangle ]\\  &  & +\,{|1\rangle }_{1}{(1-{p}_{p})}^{5}[|1100\rangle +|1001\rangle +|0110\rangle +|0011\rangle ]\\  &  & \times \,[|111000\rangle +|110001\rangle +|101010\rangle +|100011\rangle \\  &  & +\,|011100\rangle +|010101\rangle +|001110\rangle +|000111\rangle ]\}\\  &  & \times \,\{{\langle 0|}_{1}{(1-{p}_{p})}^{5}[\langle 0000|+\langle 0101|+\langle 1010|\\  &  & +\,\langle 1111|][\langle 000000|+\langle 001001|+\langle 010010|\\  &  & +\,\langle 011011|+\langle 100100|+\langle 101101|+\langle 110110|\\  &  & +\,\langle 111111|]+{\langle 1|}_{1}{(1-{p}_{p})}^{5}[\langle 1100|\\  &  & +\,\langle 1001|+\langle 0110|+\langle 0011|][\langle 111000|\\  &  & +\,\langle 110001|+\langle 101010|+\langle 100011|\\  &  & +\,\langle 011100|+\langle 010101|+\langle 001110|\\  &  & +\,\langle 000111|]+[{|0\rangle }_{1}\sqrt{{p}_{p}^{5}}{(\sqrt{1-{p}_{p}})}^{5}|0000\rangle |000000\rangle \\  &  & +\,{|1\rangle }_{1}\sqrt{{p}_{p}^{5}}|0011\rangle |111000\rangle ][{\langle 0|}_{1}\sqrt{{p}_{p}^{5}}{(\sqrt{1-{p}_{p}})}^{5}\langle 0000|\langle 000000|\\  &  & +\,{\langle 1|}_{1}\sqrt{{p}_{p}^{5}}\langle 0011|\langle 111000|]+[{|0\rangle }_{1}\sqrt{{p}_{p}^{5}}{(\sqrt{1-{p}_{p}})}^{5}|1111\rangle |111111\rangle \\  &  & +\,{|1\rangle }_{1}\sqrt{{p}_{p}^{5}}|1100\rangle |000111\rangle ][{\langle 0|}_{1}\sqrt{{p}_{p}^{5}}{(\sqrt{1-{p}_{p}})}^{5}\langle 1111|\langle 111111|\\  &  & +\,{\langle 1|}_{1}\sqrt{{p}_{p}^{5}}\langle 1100|\langle 000111|]+[{|0\rangle }_{1}\sqrt{{p}_{p}^{5}}{(\sqrt{1-{p}_{p}})}^{5}|0000\rangle |000000\rangle \\  &  & +\,{|1\rangle }_{1}\sqrt{{p}_{p}^{5}}|1100\rangle |000111\rangle ][{\langle 0|}_{1}\sqrt{{p}_{p}^{5}}{(\sqrt{1-{p}_{p}})}^{5}\langle 0000|\langle 000000|\\  &  & +\,{\langle 1|}_{1}\sqrt{{p}_{p}^{5}}\langle 1100|\langle 000111|]+[{|0\rangle }_{1}{p}_{p}^{5}|0000\rangle |000000\rangle ]\\  &  & \times \,[{\langle 0|}_{1}{p}_{p}^{5}\langle 0000|\langle 000000|]+[{|1\rangle }_{1}{p}_{p}^{5}|1100\rangle |000111\rangle ]\\  &  & \times \,[{\langle 1|}_{1}{p}_{p}^{5}\langle 1100|\langle 000111|]+[{|0\rangle }_{1}\sqrt{{p}_{p}^{5}}{(\sqrt{1-{p}_{p}})}^{5}|1111\rangle |111111\rangle \\  &  & +\,{|1\rangle }_{1}\sqrt{{p}_{p}^{5}}|0011\rangle |111000\rangle ][{\langle 0|}_{1}\sqrt{{p}_{p}^{5}}{(\sqrt{1-{p}_{p}})}^{5}\\  &  & \times \,\langle 1111|\langle 111111|+{\langle 1|}_{1}\sqrt{{p}_{p}^{5}}\langle 0011|\langle 111000|]\\  &  & +\,[{|1\rangle }_{1}{p}_{p}^{5}|0011\rangle |111000\rangle ][{\langle 1|}_{1}{p}_{p}^{5}\langle 0011|\langle 111000|]\\  &  & +\,[{|0\rangle }_{1}{p}_{p}^{5}|1111\rangle |111111\rangle ][{\langle 0|}_{1}{p}_{p}^{5}\langle 1111|\langle 111111|]\}.\end{array}$$

#### The fidelity of the output state

In the noisy environment, Alice and Bob implement measurements on qubits 2, 4 and 7, 9, *b*, respectively. The output state can be calculated as ${({\rho }_{out}^{A})}_{3568a}$ and ${({\rho }_{out}^{P})}_{3568a}$:

14 $$\begin{array}{rcl}{({\rho }_{out}^{A})}_{3568a} & = & \frac{1}{{[1+{(1-{p}_{a})}^{2}]}^{5}+2{p}_{a}^{5}{(1-{p}_{a})}^{5}+{p}_{a}^{10}}\{[|00\rangle +(1-{p}_{a}){e}^{i{\theta }_{1}}|01\rangle \\  &  & +\,(1-{p}_{a}){e}^{i{\theta }_{2}}|10\rangle +{(1-{p}_{a})}^{2}{e}^{i{\theta }_{3}}|11\rangle ][|000\rangle \\  &  & +\,(1-{p}_{a}){e}^{i{\delta }_{1}}|001\rangle +(1-{p}_{a}){e}^{i{\delta }_{2}}|010\rangle +{(1-{p}_{a})}^{2}{e}^{i{\delta }_{3}}|011\rangle \\  &  & +\,(1-{p}_{a}){e}^{i{\delta }_{4}}|100\rangle +{(1-{p}_{a})}^{2}{e}^{i{\delta }_{5}}|101\rangle +{(1-{p}_{a})}^{2}{e}^{i{\delta }_{6}}|110\rangle \\  &  & +\,{(1-{p}_{a})}^{3}{e}^{i{\delta }_{7}}|111\rangle ][\langle 00|+(1-{p}_{a}){e}^{-i{\theta }_{1}}\langle 01|\\  &  & +\,(1-{p}_{a}){e}^{-i{\theta }_{2}}\langle 10|+{(1-{p}_{a})}^{2}{e}^{-i{\theta }_{3}}\langle 11|][\langle 000|+(1-{p}_{a}){e}^{-i{\delta }_{1}}\langle 001|\\  &  & +\,(1-{p}_{a}){e}^{-i{\delta }_{2}}\langle 010|+{(1-{p}_{a})}^{2}{e}^{-i{\delta }_{3}}\langle 011|+(1-{p}_{a}){e}^{-i{\delta }_{4}}\langle 100|\\  &  & +\,{(1-{p}_{a})}^{2}{e}^{-i{\delta }_{5}}\langle 101|+{(1-{p}_{a})}^{2}{e}^{-i{\delta }_{6}}\langle 110|+{(1-{p}_{a})}^{3}{e}^{-i{\delta }_{7}}\langle 111|]\\  &  & +\,{p}_{a}^{5}{(1-{p}_{a})}^{5}[{e}^{i{\theta }_{3}}|00\rangle {e}^{i{\delta }_{7}}|111\rangle ][{e}^{-i{\theta }_{3}}\langle 00|{e}^{-i{\delta }_{7}}\langle 111|]+{p}_{a}^{5}{(1-{p}_{a})}^{5}\\  &  & \times \,[{e}^{i{\theta }_{3}}|11\rangle {e}^{i{\delta }_{7}}|000\rangle ][{e}^{-i{\theta }_{3}}\langle 11|{e}^{-i{\delta }_{7}}\langle 000|]\\  &  & +\,{p}_{a}^{10}{e}^{i{\theta }_{3}}|00\rangle {e}^{i{\delta }_{7}}|000\rangle {e}^{-i{\theta }_{3}}\langle 00|{e}^{-i{\delta }_{7}}\langle 000|\}.\end{array}$$

15 $$\begin{array}{rcl}{({\rho }_{out}^{P})}_{3568a} & = & \frac{1}{32{(1-{p}_{p})}^{10}+4{p}_{p}^{5}{(1-{p}_{p})}^{5}+2{p}_{p}^{10}}\{{(1-{p}_{p})}^{10}[|00\rangle \\  &  & +{e}^{i{\theta }_{1}}|01\rangle +{e}^{i{\theta }_{2}}|10\rangle +{e}^{i{\theta }_{3}}|11\rangle ]\\  &  & \times \,[|000\rangle +{e}^{i{\delta }_{1}}|001\rangle +{e}^{i{\delta }_{2}}|010\rangle +{e}^{i{\delta }_{3}}|011\rangle \\  &  & +\,{e}^{i{\delta }_{4}}|100\rangle +{e}^{i{\delta }_{5}}|101\rangle +{e}^{i{\delta }_{6}}|110\rangle +{e}^{i{\delta }_{7}}|111\rangle ]\\  &  & \times \,[\langle 00|+{e}^{i{\theta }_{1}}\langle 01|+{e}^{i{\theta }_{2}}\langle 10|+{e}^{i{\theta }_{3}}\langle 11|][\langle 000|\\  &  & +\,{e}^{-i{\delta }_{1}}\langle 001|+{e}^{-i{\delta }_{2}}\langle 010|+{e}^{-i{\delta }_{3}}\langle 011|+{e}^{-i{\delta }_{4}}\langle 100|\\  &  & +\,{e}^{-i{\delta }_{5}}\langle 101|+{e}^{-i{\delta }_{6}}\langle 110|+{e}^{-i{\delta }_{7}}\langle 111|]\\  &  & +\,2{p}_{p}^{5}{(1-{p}_{p})}^{5}|00\rangle |000\rangle \langle 00|\langle 000|\\  &  & +\,2{p}_{p}^{5}{(1-{p}_{p})}^{5}{e}^{i{\theta }_{3}}|11\rangle {e}^{i{\delta }_{7}}|111\rangle {e}^{-i{\theta }_{3}}\langle 11|{e}^{-i{\delta }_{7}}\langle 111|\\  &  & +\,{p}_{p}^{10}|00\rangle |000\rangle \langle 00|\langle 000|+{p}_{p}^{10}{e}^{i{\theta }_{3}}|11\rangle {e}^{i{\delta }_{7}}\\  &  & \times \,|111\rangle {e}^{-i{\theta }_{3}}\langle 11|{e}^{-i{\delta }_{7}}\langle 111|\}.\end{array}$$

If we consider the scheme in the noiseless environment, the final state is $|{\rm{\Psi }}\rangle ={|{\phi }_{A}\rangle }_{35}\otimes {|{\phi }_{B}\rangle }_{68a}$. However, in the noisy environment, the fidelity of the output state can be calculated as ${F}^{A}=\langle {\rm{\Psi }}|{\rho }_{out}^{A}|{\rm{\Psi }}\rangle$ and ${F}^{P}=\langle {\rm{\Psi }}|{\rho }_{out}^{P}|{\rm{\Psi }}\rangle$.

16 $${F}^{A}=\frac{{[4-4{p}_{a}+{{p}_{a}}^{2}]}^{2}{[4-3{p}_{a}+3{(1-{p}_{a})}^{2}+{(1-{p}_{a})}^{3}]}^{2}+2{p}_{a}^{5}{(1-{p}_{a})}^{5}+{p}_{a}^{10}}{32\{{[1+{(1-{p}_{a})}^{2}]}^{5}+2{p}_{a}^{5}{(1-{p}_{a})}^{5}+{p}_{a}^{10}\}},$$

17 $${F}^{P}=\frac{512{(1-{p}_{p})}^{10}+2{p}_{p}^{5}{(1-{p}_{p})}^{5}+{p}_{p}^{10}}{512{(1-{p}_{p})}^{10}+64{p}_{p}^{5}{(1-{p}_{p})}^{5}+32{p}_{p}^{10}}.$$

**Figure 3:**
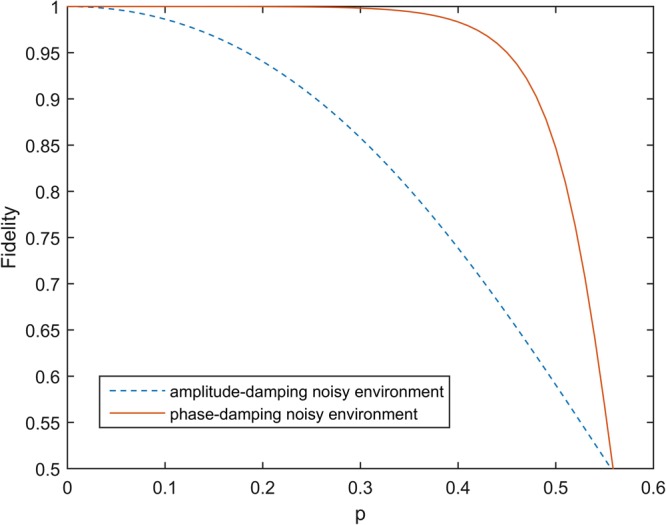
The relationship between fidelity and decoherence rate. In the amplitude-damping and phase-damping noisy environment, we assume that ${p}_{a}={p}_{p}=p$.

The fidelity is at most 1 by definition and 0.5 is automatically achieved by outputting a completely mixed state^26^. By calculating the fidelities of the output states, we conclude that fidelity depends on the decoherence rate. In amplitude-damping and phase-damping noisy environments, we assume that ${p}_{a}={p}_{p}=p$. The diagram of fidelity changes with the decoherence rate given in Fig. 3. We can see that the fidelities ${F}^{A},{F}^{P}$ of the output states decreases as the decoherence rate increases.

## Discussions

First, we give a summary of this scheme, including the necessary operations, classical communication costs (CCCs) and efficiency. Second, we give some discussions with other schemes^18–21^. Last, the conclusions are described.

In this paper, we construct the quantum channel at first. The necessary operations have six *H* operations and ten *CNOT* operations. Quantum gate operations are critical part of quantum computation and quantum communications. Many experimental results^27–31^ have been proposed for quantum operations implementation. For example, in 2018, Rosenblum *et al*.^27^ realized a *CNOT* gate between two multiphoton qubits in two microwave cavities. They coupled two encoded qubits together through a transmon, which is driven by an RF pump to apple the gate within 190 ns. This is two orders of magnitude shorter than the decoherence time of the transmon, enabling a high-fidelity gate operation. Zajac *et al*.^28^ demonstrate an efficient resonantly driven *CNOT* gate for electron spins in silicon. The single-qubit rotations can be achieved by their platform with fidelities greater than 99%, as verified by randomized benchmarking. They used the *CNOT* gate to generate a Bell state with 78% fidelity. According to the relevant experimental results, the implementation of the *H* and *CNOT* operations can be completed by using the existing technology. Furthermore, we can realize the construction of the eleven-qubit entangled state.

In our ACBRSP scheme, no auxiliary operations are needed. The CCCs are generated when three participants send their measurement results through the classical channel. In the scheme, the measurement results can be transmitted directly to others via broadcast, since it does not contain the information about the prepared state. Thus, the CCCs are 6 bits. The efficiency^32^ of the scheme can be calculated as

18 $$\eta ={q}_{s}/({q}_{u}+{b}_{t})=5/17=29.41 \% ,$$

where ${q}_{s}$ denotes the number of qubits that consist of the quantum information to be prepared, ${q}_{u}$ is the number of the qubits that is used as the quantum channel and ${b}_{t}$ is the classical bits transmitted.

We give some discussions with the other ACBRSP schemes^18–21^. The results are shown in Table 1. Firstly, in refs^18,19,21^, these schemes are asymmetrical bidirectional remote preparation of an arbitrary single- and two-qubit state. The scheme in ref.^20^ is asymmetrical bidirectional remote preparation of single- and four-qubit state, but in fact they only transmit the single- and two-qubit state and the receiver needs two local auxiliary qubits and auxiliary operations to recovery four-qubit state. However, in our ACBRSP scheme, we prepare a two- and three-qubit equatorial state. Secondly, we construct the quantum channel by using *H* operations and *CNOT* operations. Neither of them gave the process of the construction of quantum channel. Thirdly, in refs^19–21^, these schemes need the auxiliary qubits and auxiliary operations to complete the preparation task, while our scheme and the scheme in ref.^18^ do not need. Fourthly, the CCCs of our scheme are 6 bits, which is less than that in refs^20,21^. Finally, the efficiency of our scheme is higher than that of other schemes.

**Table 1:** Discussions with other ACBRSP schemes.

Scheme	Quantum Channel	Auxiliary Qubits	Auxiliary Operations	CCCs	Efficiency
ref.^20^	Ten-Qubit Entangled State	5	7 *CNOTs*	8	13.04%
ref.^18^	Seven-Qubit Entangled State	0	0	5	25%
ref.^21^	Seven-Qubit Entangled State	2	2 *CNOTs*	7	18.75%
	Seven-Qubit Entangled State	1	1 *CNOT*	7	20%
ref.^19^	Four-Qubit Entangled State + EPR	1	1 *CNOT*	6	23.08%
Ours	Eleven-Qubit Entangled State	0	0	6	29.41%

Where CCCs is short for classical communication cost.

In the future, we hope the scheme can play a facilitating role in quantum network communication. The point-to-point quantum communication must be turned to the multi-party quantum network communication. These have a wide range of research meanings in some network structures^33–36^. As regards the quantum networks, the feasibility and construction have been fully verified theoretically^37,38^. Our scheme do not need the auxiliary resources and have relatively high efficiency, so it can be easily incorporated into the design of quantum network communication.

## Conclusions

In summary, we propose a novel ACBRSP scheme. Alice prepares an arbitrary two-qubit equatorial state for Bob and Bob prepares an arbitrary three-qubit equatorial state for Alice simultaneously by using the eleven-qubit entangled state as the quantum channel. The quantum channel is constructed at first. Moreover, Alice and Bob can recover the prepared state determinately. Then, our scheme are considered in the noisy environment (amplitude-damping and phase-damping noisy environment) and the fidelities of the output states are calculated. In the end, we give some discussions with other ACBRSP schemes and the results show that our scheme is effective.

Specifically, an arbitrary two-qubit state and three-qubit state can be prepared separately in either direction. Moreover, using the quantum channel we selected, in addition to completing the content presented in this paper, Alice can also prepare a three-qubit equatorial state for Bob, and Bob prepares a two-qubit equatorial state for Alice. Furthermore, according to the construction of the quantum channel, we can see that whether it is the products of five $|{{\rm{\Phi }}}^{+}\rangle$ or products of five $|{{\rm{\Psi }}}^{+}\rangle$ states. One can partition these products into the product of any two Bell states and the product of the remaining three Bell states. The part can be used to prepare the corresponding multi-qubit state in either direction.

Our scheme does not only allow the preparation of the equatorial state. Also, since the quantum channel is suitable for processing the bidirectional transmission of quantum states. There is no strict restriction on the type of quantum states. For example, the scheme can also be used for the bidirectional preparation of general or other special states. We will further study this part of the contents.

## Supplementary information

Supplementary information of Asymmetric controlled bidirectional remote preparation of two- and three-qubit equatorial state
