# Supplementary material for: Asymmetric controlled bidirectional remote preparation of two- and three-qubit equatorial state
Source: Sci Rep. 2019 Feb 14;9:2081. doi: 10.1038/s41598-018-37957-x (PMC6376120; doi:10.1038/s41598-018-37957-x)
Supplement: Supplementary file 1 — Supplementary information of Asymmetric controlled bidirectional remote preparation of two- and three-qubit equatorial state [file 41598_2018_37957_MOESM1_ESM.pdf]

Asymmetric controlled bidirectional remote preparation of two- and three-qubit equatorial state

Yi-Ru Sun[1], Xiu-Bo Chen[1,2,*], Gang Xu[1] , Kai-Guo Yuan[1] & Yi-Xian Yang [2,1]

Supplementary information in Appendix

Table. A1 The whole recovery operations of Alice and Bob

Charlie's MR	Alice's MR	Bob's MR	$U_6 \otimes U_8 \otimes U_a$	$U_3 \otimes U_5$
$\|0\rangle_1$	$\|A_0\rangle_{24}$	$\|B_0\rangle_{79b}$	$I_6 \otimes I_8 \otimes I_a$	$I_3 \otimes I_5$
		$\|B_1\rangle_{79b}$	$I_6 \otimes I_8 \otimes Z_a$	
		$\|B_2\rangle_{79b}$	$I_6 \otimes I_8 \otimes P(\pi/2)_a$	
		$\|B_3\rangle_{79b}$	$I_6 \otimes Z_8 \otimes P(3\pi/2)_a$	
		$\|B_4\rangle_{79b}$	$Z_6 \otimes P(\pi/2)_8 \otimes P(\pi/4)_a$	
		$\|B_5\rangle_{79b}$	$Z_6 \otimes P(\pi/2)_8 \otimes P(5\pi/4)_a$	
		$\|B_6\rangle_{79b}$	$Z_6 \otimes P(3\pi/2)_8 \otimes P(3\pi/4)_a$	
		$\|B_7\rangle_{79b}$	$Z_6 \otimes P(3\pi/2)_8 \otimes P(7\pi/4)_a$	
$\|0\rangle_1$	$\|A_1\rangle_{24}$	$\|B_0\rangle_{79b}$	$I_6 \otimes I_8 \otimes I_a$	$I_3 \otimes Z_5$
		$\|B_1\rangle_{79b}$	$I_6 \otimes I_8 \otimes Z_a$	
		$\|B_2\rangle_{79b}$	$I_6 \otimes I_8 \otimes P(\pi/2)_a$	
		$\|B_3\rangle_{79b}$	$I_6 \otimes Z_8 \otimes P(3\pi/2)_a$	
		$\|B_4\rangle_{79b}$	$Z_6 \otimes P(\pi/2)_8 \otimes P(\pi/4)_a$	
		$\|B_5\rangle_{79b}$	$Z_6 \otimes P(\pi/2)_8 \otimes P(5\pi/4)_a$	
		$\|B_6\rangle_{79b}$	$Z_6 \otimes P(3\pi/2)_8 \otimes P(3\pi/4)_a$	
		$\|B_7\rangle_{79b}$	$Z_6 \otimes P(3\pi/2)_8 \otimes P(7\pi/4)_a$	
		$\|B_0\rangle_{79b}$	$I_6 \otimes I_8 \otimes I_a$	
		$\|B_1\rangle_{79b}$	$I_6 \otimes I_8 \otimes Z_a$	

[1]Information Security Center, State Key Laboratory of Networking and Switching Technology, Beijing University of Posts and Telecommunications, Beijing 100876, China. [2] GuiZhou University, Guizhou Provincial Key Laboratory of Public Big Data, Guizhou Guiyang, 550025, China. Correspondence and requests for materials should be addressed to Xiu-Bo Chen (email: flyover100@163.com)

$\vert 0\rangle_1$	$\vert A_2\rangle_{24}$	$\vert B_2\rangle_{79b}$	$I_6 \otimes I_8 \otimes P(\pi/2)_a$	$Z_3 \otimes P(\pi/2)_5$
		$\vert B_3\rangle_{79b}$	$I_6 \otimes Z_8 \otimes P(3\pi/2)_a$	
		$\vert B_4\rangle_{79b}$	$Z_6 \otimes P(\pi/2)_8 \otimes P(\pi/4)_a$	
		$\vert B_5\rangle_{79b}$	$Z_6 \otimes P(\pi/2)_8 \otimes P(5\pi/4)_a$	
		$\vert B_6\rangle_{79b}$	$Z_6 \otimes P(3\pi/2)_8 \otimes P(3\pi/4)_a$	
		$\vert B_7\rangle_{79b}$	$Z_6 \otimes P(3\pi/2)_8 \otimes P(7\pi/4)_a$	
$\vert 0\rangle_1$	$\vert A_3\rangle_{24}$	$\vert B_0\rangle_{79b}$	$I_6 \otimes I_8 \otimes I_a$	$Z_3 \otimes P(3\pi/2)_5$
		$\vert B_1\rangle_{79b}$	$I_6 \otimes I_8 \otimes Z_a$	
		$\vert B_2\rangle_{79b}$	$I_6 \otimes I_8 \otimes P(\pi/2)_a$	
		$\vert B_3\rangle_{79b}$	$I_6 \otimes Z_8 \otimes P(3\pi/2)_a$	
		$\vert B_4\rangle_{79b}$	$Z_6 \otimes P(\pi/2)_8 \otimes P(\pi/4)_a$	
		$\vert B_5\rangle_{79b}$	$Z_6 \otimes P(\pi/2)_8 \otimes P(5\pi/4)_a$	
		$\vert B_6\rangle_{79b}$	$Z_6 \otimes P(3\pi/2)_8 \otimes P(3\pi/4)_a$	
		$\vert B_7\rangle_{79b}$	$Z_6 \otimes P(3\pi/2)_8 \otimes P(7\pi/4)_a$	
$\vert 1\rangle_1$	$\vert A_0\rangle_{24}$	$\vert B_0\rangle_{79b}$	$X_6 \otimes X_8 \otimes X_a$	$X_3 \otimes X_5$
		$\vert B_1\rangle_{79b}$	$X_6 \otimes X_8 \otimes iY_a$	
		$\vert B_2\rangle_{79b}$	$X_6 \otimes X_8 \otimes P(\pi/2)_a X_a$	
		$\vert B_3\rangle_{79b}$	$X_6 \otimes iY_8 \otimes P(3\pi/2)_a X_a$	
		$\vert B_4\rangle_{79b}$	$iY_6 \otimes P(\pi/2)_8 X_8 \otimes P(\pi/4)_a X_a$	
		$\vert B_5\rangle_{79b}$	$iY_6 \otimes P(\pi/2)_8 X_8 \otimes P(5\pi/4)_a X_a$	
		$\vert B_6\rangle_{79b}$	$iY_6 \otimes P(3\pi/2)_8 X_8 \otimes P(3\pi/4)_a X_a$	
		$\vert B_7\rangle_{79b}$	$iY_6 \otimes P(3\pi/2)_8 X_8 \otimes P(7\pi/4)_a X_a$	
$\vert 1\rangle_1$	$\vert A_1\rangle_{24}$	$\vert B_0\rangle_{79b}$	$X_6 \otimes X_8 \otimes X_a$	$X_3 \otimes X_5$
		$\vert B_1\rangle_{79b}$	$X_6 \otimes X_8 \otimes iY_a$	
		$\vert B_2\rangle_{79b}$	$X_6 \otimes X_8 \otimes P(\pi/2)_a X_a$	
		$\vert B_3\rangle_{79b}$	$X_6 \otimes iY_8 \otimes P(3\pi/2)_a X_a$	
		$\vert B_4\rangle_{79b}$	$iY_6 \otimes P(\pi/2)_8 X_8 \otimes P(\pi/4)_a X_a$	

		$\vert B_5\rangle_{79b}$	$iY_6\otimes P(\pi/2)_8 X_8\otimes P(5\pi/4)_a X_a$	
		$\vert B_6\rangle_{79b}$	$iY_6\otimes P(3\pi/2)_8 X_8\otimes P(3\pi/4)_a X_a$	
		$\vert B_7\rangle_{79b}$	$iY_6\otimes P(3\pi/2)_8 X_8\otimes P(7\pi/4)_a X_a$	
$\vert 1\rangle_1$	$\vert A_2\rangle_{24}$	$\vert B_0\rangle_{79b}$	$X_6\otimes X_8\otimes X_a$	$iY_3\otimes P(\pi/2)_5 X_5$
		$\vert B_1\rangle_{79b}$	$X_6\otimes X_8\otimes iY_a$	
		$\vert B_2\rangle_{79b}$	$X_6\otimes X_8\otimes P(\pi/2)_a X_a$	
		$\vert B_3\rangle_{79b}$	$X_6\otimes iY_8\otimes P(3\pi/2)_a X_a$	
		$\vert B_4\rangle_{79b}$	$iY_6\otimes P(\pi/2)_8 X_8\otimes P(\pi/4)_a X_a$	
		$\vert B_5\rangle_{79b}$	$iY_6\otimes P(\pi/2)_8 X_8\otimes P(5\pi/4)_a X_a$	
		$\vert B_6\rangle_{79b}$	$iY_6\otimes P(3\pi/2)_8 X_8\otimes P(3\pi/4)_a X_a$	
		$\vert B_7\rangle_{79b}$	$iY_6\otimes P(3\pi/2)_8 X_8\otimes P(7\pi/4)_a X_a$	
$\vert 1\rangle_1$	$\vert A_3\rangle_{24}$	$\vert B_0\rangle_{79b}$	$X_6\otimes X_8\otimes X_a$	$iY_3\otimes P(3\pi/2)_5 X_5$
		$\vert B_1\rangle_{79b}$	$X_6\otimes X_8\otimes iY_a$	
		$\vert B_2\rangle_{79b}$	$X_6\otimes X_8\otimes P(\pi/2)_a X_a$	
		$\vert B_3\rangle_{79b}$	$X_6\otimes iY_8\otimes P(3\pi/2)_a X_a$	
		$\vert B_4\rangle_{79b}$	$iY_6\otimes P(\pi/2)_8 X_8\otimes P(\pi/4)_a X_a$	
		$\vert B_5\rangle_{79b}$	$iY_6\otimes P(\pi/2)_8 X_8\otimes P(5\pi/4)_a X_a$	
		$\vert B_6\rangle_{79b}$	$iY_6\otimes P(3\pi/2)_8 X_8\otimes P(3\pi/4)_a X_a$	
		$\vert B_7\rangle_{79b}$	$iY_6\otimes P(3\pi/2)_8 X_8\otimes P(7\pi/4)_a X_a$	

Here, MR is short for the measurement result and the $U_6\otimes U_8\otimes U_a$, $U_3\otimes U_5$ are the recovery operations of Alice and Bob, respectively. *I*, *X*, *Y*, *Z* are the *Pauli* matrix and $P(\theta)=\begin{pmatrix}1 & 0\\ 0 & e^{i\theta}\end{pmatrix}, 0\le\theta\le 2\pi$.
